# On the Performance of Thin-Walled Crash Boxes Joined by Forming

**DOI:** 10.3390/ma11071118

**Published:** 2018-06-29

**Authors:** Diogo F. M. Silva, Carlos M. A. Silva, Ivo M. F. Bragança, Chris V. Nielsen, Luis M. Alves, Paulo A. F. Martins

**Affiliations:** 1 Instituto de Engenharia Mecânica, Instituto Superior Técnico, Universidade de Lisboa, Av. Rovisco Pais, 1049-001 Lisboa, Portugal; diogogjmsilva@tecnico.ulisboa.pt (D.F.M.S.); carlos.alves.silva@tecnico.ulisboa.pt (C.M.A.S.); luisalves@tecnico.ulisboa.pt (L.M.A.); 2 Instituto Superior de Engenharia de Lisboa, Rua Conselheiro Emídio Navarro, 1959-007 Lisboa, Portugal; ibraganca@dem.isel.pt; 3 Department of Mechanical Engineering, Technical University of Denmark, 2800 Kgs. Lyngby, Denmark; cvni@mek.dtu.dk

**Keywords:** crash boxes, joining by forming, resistance spot-welding, crashworthiness

## Abstract

A new joining by forming process that combines lancing and shearing with sheet-bulk compression is utilized to assemble thin-walled crash boxes utilized as energy absorbers. Process design and fabrication of the new crash boxes are analyzed by finite elements and experimentation. Axial crush tests were performed to compare the overall crashworthiness performance of the new crash boxes against that of conventional crash boxes assembled by resistance spot-welding. Results show that the joining process is a good alternative to resistance spot-welding because the new crash boxes can absorb the same crushing energy, and because the new process helps to overcome typical manufacturing problems of welding.

## 1. Introduction

An energy absorber is an important element of a vehicle because it protects the lives of passengers by managing the absorption of energy and collapse of its structure during an accident. One of the strategies currently employed by vehicle manufacturers to meet the increasing requirements on safety and the diminishing weight targets is the utilization of energy absorbers made of high-strength materials in lightweight body structures. However, the search for more effective energy absorbers is wider and includes new geometries and materials with the potential to enhance the crashworthiness performance of the structures under different types of loading. These trends are comprehensively discussed in two recently published state-of-the-art reviews on energy absorbers [1,2].

The search for new processes to manufacture energy absorbers has so far received little attention. Most of the publications in the field make use of energy absorbers produced by conventional extrusion [3,4] or by combination of forming and welding [5,6] (or, adhesive bonding) with or without hydroforming [7]. However, the design of energy absorbers with higher performance and wider applicability than the existing ones requires the development of new fabrication processes that allows for a combination of dissimilar materials with different thicknesses and types of surface coatings, as well as a combination of adhesives.

Joining by forming [8] can also be successfully utilized to fabricate efficient, low-cost, energy absorbers. Lee et al. [9], for example, made use of self-piercing riveting to assemble thin-walled crash boxes with double hat-shaped sections made from steel and aluminum formed panels. They analyzed the overall crashworthiness performance and concluded that the energy absorbed by the self-piercing riveted crash boxes is higher than that of the adhesive-bonded. Gronostajski and Polak [10] utilized two different clinching processes to assemble thin-walled crash boxes with double hat-shaped sections made of steel formed panels and concluded that clinching can be successfully applied to fabricate energy absorbers.

Table 1 summarizes the main advantages and disadvantages of the fabrication processes that are commonly utilized to assemble crash boxes with top-hat and double-hat shaped sections made from individual formed panels.

**Figure 1:**
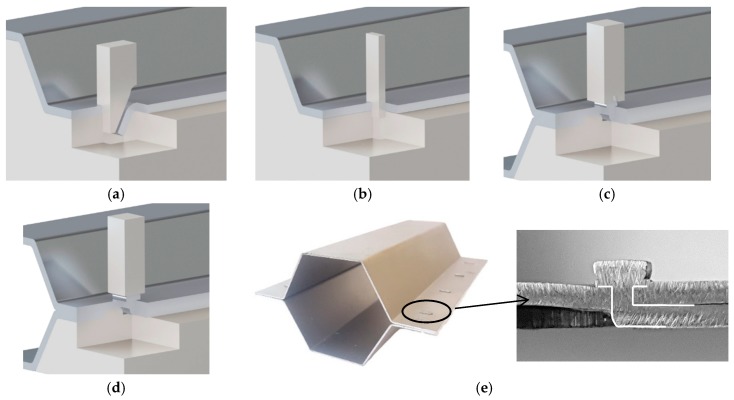
New fabrication process to assemble the individual formed panels of thin-walled crash boxes with double-hat shaped sections: (**a**) lancing (shearing and bending) of the tenon; (**b**) shearing of the mortise; (**c**) sheet-bulk compression of the tenon with a tapered punch; (**d**) mechanical locking by sheet-bulk compression of the tenon with a flat punch; (**e**) cross-section of a “mortise-and-tenon” joint. Note: the lancing in (**a**) is upside-down in order to replicate the press movement.

This paper is focused on the assembly of thin-walled crash boxes with double-hat shaped sections made from individual formed panels. The aim and objective is to present a new fabrication process that combines lancing of the tenons, in which specific areas of the panels are sheared and bent in a single press operation (Figure 1a), shearing of the mortises, in which holes are cut out of the opposite panels (Figure 1b), and sheet-bulk compression of the tenons during which the two panels are assembled by means of “mortise-and-tenon” joints placed along the flanges (Figure 1c,d).

The new fabrication process draws from two previous investigations on joining by forming in which “mortise-and-tenon” joints were successfully utilized to fix two sheets longitudinally in position by sheet-bulk compression. The two sheets were joined either perpendicular [11] or parallel [12] to each other. The reason behind the utilization of “mortise-and-tenon” joints as an alternative to resistance spot-welded or adhesive bonded joints is because they offer the same advantages of self-piercing riveted and clinched joints (refer to Table 1) without having the constraints related to organic coatings and lubricants. Moreover, the “mortise-and-tenon” joints have the ability to be easily combined with adhesives in order to increase stiffness and are also capable of ensuring higher-peel strength than self-piercing riveted and clinched joints due to the protrusion of the flat-shaped surface heads of the tenons above the adjacent sheet panels, after compression.

The main challenge of applying the “mortise-and-tenon” joint concept in the assembly of crash boxes (Figure 1e) derives from the need to compress thin-walled formed panels in the direction perpendicular to thickness. However, the authors solved the problem by introducing a novel two-stage variant of the sheet-bulk compression process that makes uses of a tapered heading punch in the first stage (Figure 1c), and a flat heading punch in the second stage (Figure 1d). The tapered heading punch ensures a better balance of material displacement and diminishes the risk of buckling during the initial compression of the panels. The flat heading punch ensures the mechanical locking of the two individual panels.

The crash boxes assembled with the new proposed “mortise-and-tenon” joint concept are subjected to static and dynamic axial crushing and its overall crashworthiness performance is compared against that of resistance spot-welded. Results show that the new crash boxes are a good alternative to those assembled by resistance spot-welding.

## 2. Materials and Methods

### 2.1. Mechanical Characterization

The new proposed joining by forming process can easily assemble thin-walled crash boxes with individual panels made from dissimilar materials with different thicknesses. However, it was decided to select a single high-strength low alloy steel (HSLA 340) with 1 mm thickness and 7 µm thickness galvanized coating to ensure a fair comparison of the crashworthiness performance of the new crash boxes against those assembled by resistance spot-welding.

The mechanical characterization of the HSLA 340 steel was performed by means of stack compression tests [13] due to its capability to characterize the material stress response up to the large strains that were found in the compression of the flat-shaped surface heads of the tenons. The stack compression tests were carried out at room temperature in a hydraulic testing machine (Instron SATEC 1200 kN, Norwood, MA, USA) with a cross-head velocity of 10 mm/min and made use of multi-layer cylinder test specimens that were assembled by piling up 10 circular disks with 10 mm diameter cut out from the supplied sheets by wire-electro discharge machining (wire-EDM).

### 2.2. Fabrication of the Crash Boxes

The thin-walled crash boxes with double hat-shaped sections were made from two individual formed panels that were assembled by sheet-bulk compression with “mortise-and-tenon” joints placed every 40 mm along their flanges. Table 2 provides the geometry of the crash boxes and a schematic detail of the new proposed joining by forming process.

Conventional thin-walled crash boxes assembled by resistance spot welding were included in the experimental work plan for reference purposes. The welding parameters were selected by finite element modelling (refer to Section 3.2) and the joints consisted of around 5.4 mm diameter spots, positioned similarly along the flange as the “mortise-and-tenon” joints.

### 2.3. Axial Crush Tests

The crash boxes were tested for quasi-static and dynamic axial crushing at room temperature. The quasi-static crush tests were performed in the hydraulic testing machine (Instron SATEC 1200 kN) that had been used in the mechanical characterization of the material. A cross-head velocity v=10 mm/min was utilized, and the tests were stopped after reaching a prescribed crushing distance of 55 mm (approximately 1/3 of the initial length of the crash boxes).

**Figure 2:**
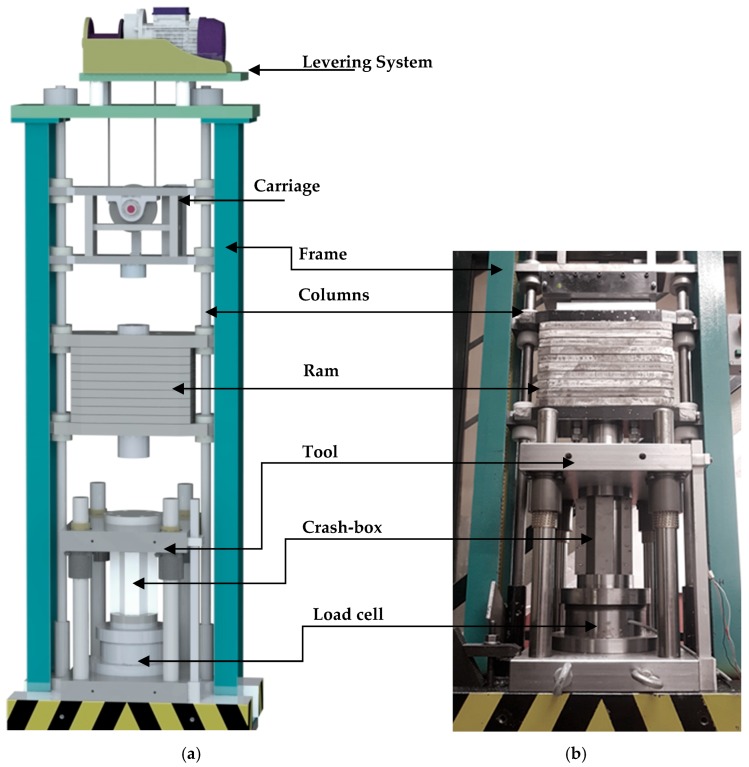
The drop weight testing machine: (**a**) schematic representation and identification of its main components; (**b**) photograph of the tool utilized in the crush tests.

The dynamic crush tests were performed in a drop weight testing machine that was designed and fabricated by the authors. The machine and its main components are schematically shown in Figure 2a. The mass M and drop height H of the falling ram can be adjusted up to maximum values of 250 kg and 5 m, respectively.

The tool utilized in the quasi-static and dynamic crush tests is shown in Figure 2b. The crash boxes were placed centrally in the tool, without any further support and were subsequently compressed between the top and bottom flat platens. The tool was instrumented with a load cell based on traditional strain gauge technology in full wheatstone bridge with a capacity of 500 kN, a nominal sensitivity of 1 mV/V, and an accuracy class of 0.7. The load cell was connected to a signal amplifier unit (Vishay 2310B) and a personal computer data logging system based on a DAQ card (National Instruments, PCI-6115, Austin, TX, USA). The displacement transducer is a commercial linear variable differential transformer (Solartron LVDT AC15, Farnborough, UK). A special purpose LabView based software was designed to acquire and store the experimental data from both the load cell and the displacement transducer.

Table 3 gives a summary of the main operating conditions utilized in the quasi-static and the dynamic axial crush tests.

### 2.4. Finite Element Modelling

The assembly of the individual panels of the thin-walled crash boxes by means of sheet-bulk compression with “mortise-and-tenon” joints was simulated with the finite element computer program I-form. The computer program was developed by the authors and is based on the irreducible finite element formulation,

(1) $$\Pi =\int_{V}\bar{\sigma }\dot{\bar{\varepsilon }}dV+\frac{1}{2}K\int_{V}{\dot{\varepsilon }}_{V}^{2}dV-\int_{S_{T}}T_{i}u_{i}dS+\int_{S_{f}}\left(\int_{0}^{\left|u_{r}\right|}\tau_{f}du_{r}\right)dS,$$

where, the symbol $\bar{\sigma }$ is the effective stress, $\dot{\bar{\varepsilon }}$ is the effective strain rate, ${\dot{\varepsilon }}_{V}$ is the volumetric strain rate, K is a large positive constant imposing the incompressibility of volume V, S is the surface under consideration, $T_{i}$ and $u_{i}$ are the surface tractions and velocities on surface $S_{T}$, $\tau_{f}$ and $u_{r}$ are the friction shear stress and the relative velocity on the contact interface $S_{f}$ between the material and tooling. Further details on the computer program with special emphasis to contact and frictional sliding between rigid and deformable objects are available in Reference [14].

The numerical simulation made use of two-dimensional plane strain deformation models and the cross section of the tenons and mortises were discretized by means of approximately 1000 quadrilateral elements. The tools were modelled as rigid objects and their geometries were discretized by means of linear contact-friction elements.

The assembly of the individual panels of the thin-walled crash boxes by means of resistance spot-welding was simulated with the commercial finite element computer program SORPAS [15], which is based on an electro-thermo-mechanical formulation as described in details in [14]. The mechanical formulation follows Equation (1). Solution of the electrical potential Φ is based on integration of Laplace’s equation, which in variational form can be written as

(2) $$\int_{V}\Phi_{,i}\delta \Phi_{,i}dV=0,$$

where subscripts indicate spatial derivatives. The current density, calculated from the derivative of the potential and electrical resistivity, is used in the calculation of Joule heating during the welding process. The following variational equation governs the thermal solution for the temperature field T,

(3) $$\int_{V}kT_{,i}\delta T_{,i}dV+\int_{V}\rho c\dot{T}\delta TdV-\int_{V}\dot{q}\delta TdV-\int_{S}kT_{,n}dS=0$$

The first term is related to heat conduction through the conductivity k, and the second term is related to the temperature rate $\dot{T}$ with material properties being mass density ρ and heat capacity c. Joule heating and heating from plastic work are included in the heat generation rate per volume $\dot{q}$ in the third term. Finally, the last term includes heat generation and loss along surfaces stemming from frictional heating, convection and radiation. The subscript in the last term refers to spatial derivative along a surface normal. Further details like treatment of contact conditions are available in Reference [14].

## 3. Results

### 3.1. Mechanical Characterization

**Figure 3:**
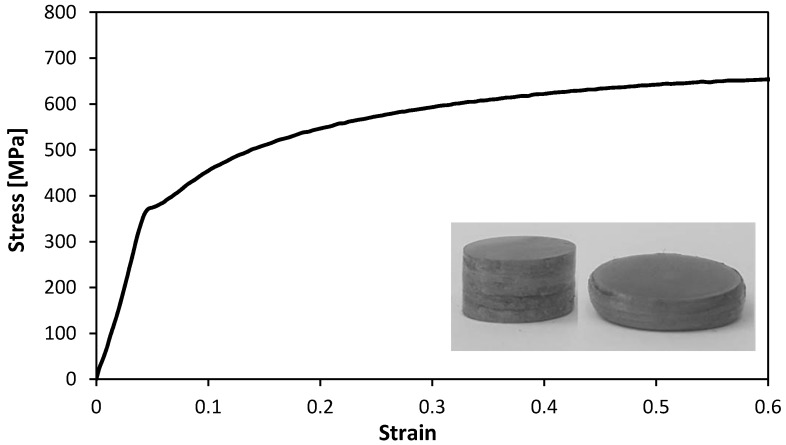
Stress-strain curve of the high-strength low alloy (HSLA) 340 steel obtained from stack compression tests.

The stress-strain curve of the HSLA 340 steel obtained from the stack compression tests is shown in Figure 3. The enclosed photograph shows the multi-layer cylinder test specimens that were assembled by piling up 10 circular disks before and after compression. The stress-strain curve is utilized in the finite element simulation of the assembly of thin-walled crash boxes by sheet-bulk compression with “mortise-and-tenon” joints.

### 3.2. Finite Element Modelling and Experimentation of the Fabrication Process

**Figure 4:**
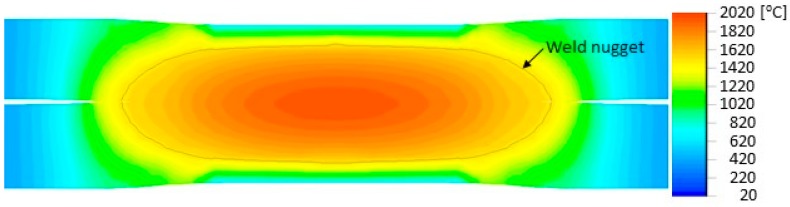
Distribution of simulated peak temperature in resistance spot-welding together with identification of the weld nugget.

The reference joining process was simulated by the commercial software SORPAS [15]. The simulation result is shown in Figure 4 in terms of process peak temperature and identification of weld nugget. The simulation was based on the parameters given in Table 2. The simulation reveals a weld nugget with a diameter of 5.4 mm across the interface and a proper penetration into both of the two HSLA 340 sheets.

**Figure 5:**
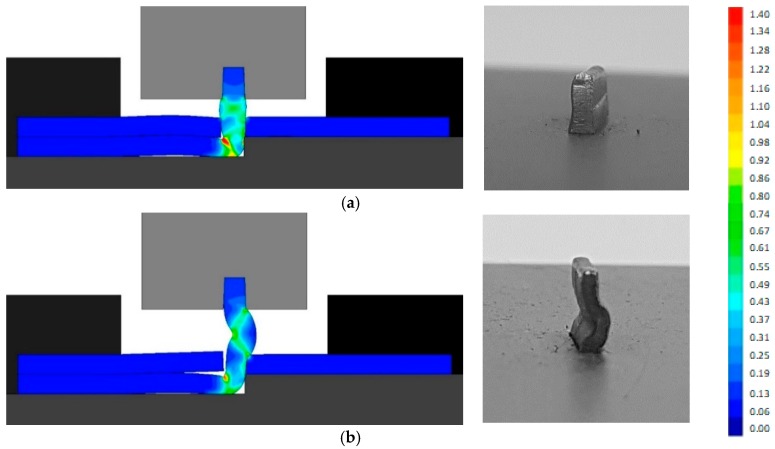
Assembling the individual formed panels of the thin-walled crash boxes: (**a**) effective strain distribution at the end of the first stage of the sheet-bulk compression of a tenon with a free length h=4.5 mm; (**b**) effective strain distribution at the end of the first stage of the sheet-bulk compression of a tenon with a free length h=6 mm.

The assembly of the thin-walled crash boxes by the novel sheet-bulk compression process with “mortise-and-tenon” joints required the tenons to be cut and bent out of the panels by lancing (Figure 1a), and subsequently compressed in the direction perpendicular to thickness (Figure 1c,d). Figure 5a,b show the finite element computed distribution of effective strain in the tenons at the end of the first sheet-bulk compression stage. Two different process operating conditions (Table 2) are shown: a tenon with a free length h=4.5 mm that is successfully compressed by a tapered heading punch (Figure 5a) and a tenon with a free length h=6 mm that fails by buckling (Figure 5b).

Successfully compressed tenons are needed for the second stage of the sheet-bulk compression, during which a flat heading punch assembles the crash boxes by mechanically locking the individual formed panels to each other. 

**Figure 6:**
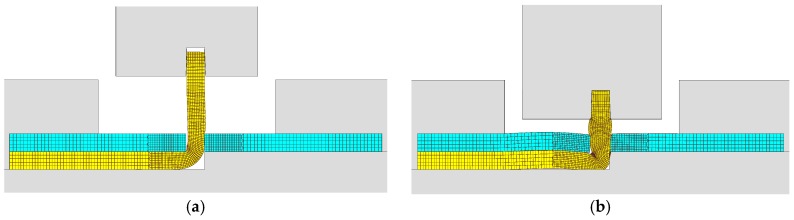

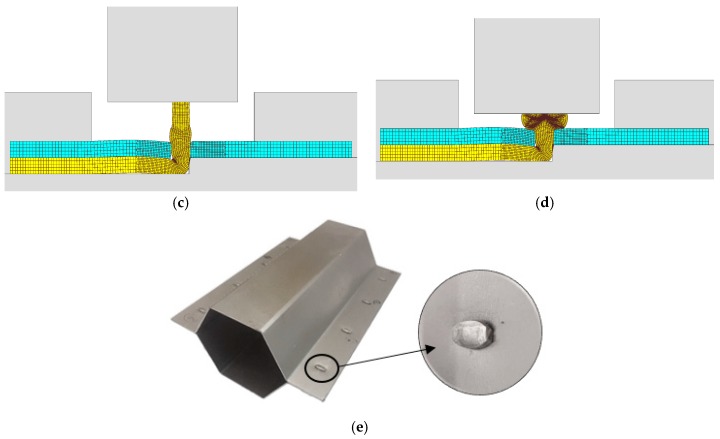
Fabrication of the crash boxes by sheet-bulk compression with “mortise-and-tenon” joints (h=4.5 mm): (**a**) finite element mesh at the beginning of the first stage; (**b**) computed finite element mesh at the end of the first stage; (**c**) computed finite element mesh at the beginning of the second stage; (**d**) computed finite element mesh at the end of the second stage; (**e**) crash box with a detail showing the flat-shaped surface head of a compressed tenon.

Figure 6 shows finite element deformed meshes at different instants of the crash box assembly by means of sheet-bulk compression with “mortise-and-tenon” joints. The case included in the figure consists of a bent tenon with a free length h=4.5 mm and corresponds to the working conditions that were utilized to fabricate all the crash boxes with “mortise-and-tenon” joints that were subjected to the axial crush tests.

As seen in Figure 6a,b, the tapered heading punch prevents the collapse by buckling at the early stages of deformation. The flat heading punch (Figure 6c,d) produces the flat-shaped surface heads (Figure 6e) that will lock the panels to each other and assemble the crash box.

**Figure 7:**
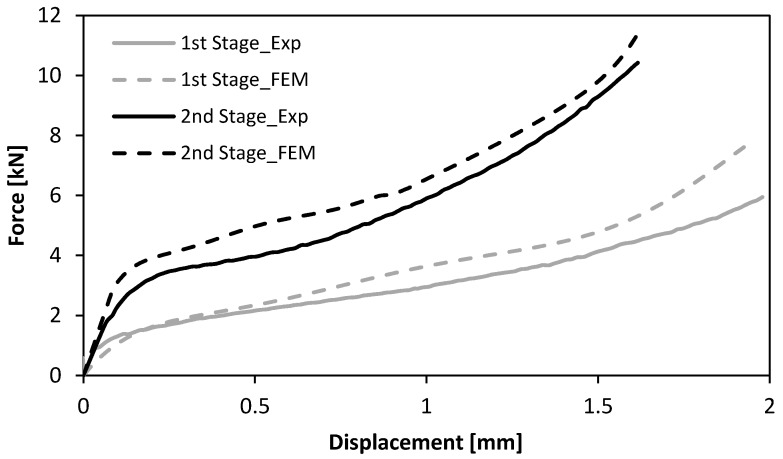
Experimental and numerical evolution of the force with displacement for the first and second stages of the sheet-bulk compression of tenons with h=4.5 mm.

Figure 7 shows the experimental and finite element computed evolution of the force with displacement for the first and second stages of the sheet-bulk compression with “mortise-and-tenon” joints. The agreement is good and allows estimating the energy required to perform the first and second stages of the new proposed joining by forming process.

### 3.3. Axial Crush Tests

**Figure 8:**
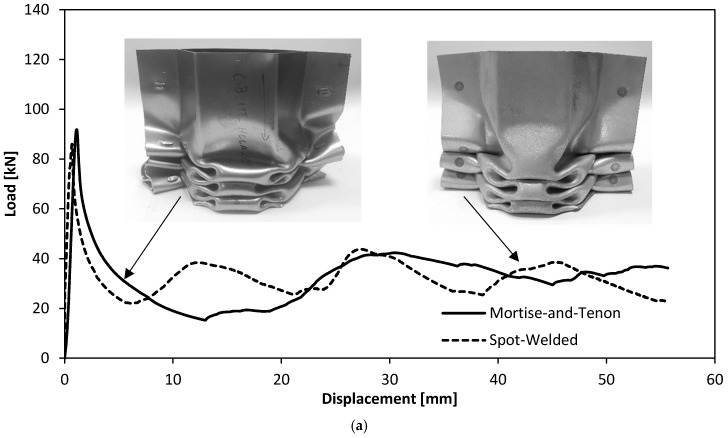

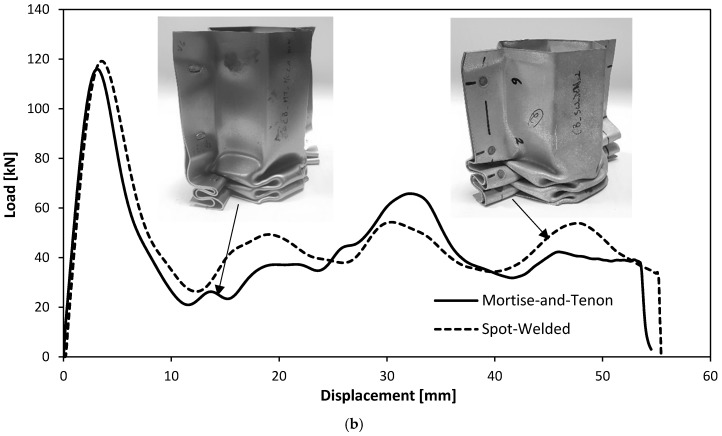
Experimental evolution of the force with displacement for the axial crush tests of thin-walled crash boxes with double-hat section assembled by sheet-bulk compression with “mortise-and-tenon” joints and by resistance spot-welding: (**a**) quasi-static tests; (**b**) dynamic tests.

Figure 8a shows the force-displacement curves for the quasi-static axial crush tests of the thin-walled crash boxes with double-hat section assembled by sheet-bulk compression with “mortise-and-tenon” joints and by resistance spot-welding. As seen, the force increases steeply up to a peak value where the first fold is formed (initiation of collapse). Then, the force decreases and the subsequent folds, corresponding to the oscillations of the force-displacement curve, are triggered for smaller local force peaks.

Figure 8b shows the force-displacement curves for the dynamic axial crush tests of the two types of crash boxes. The tests were performed in the drop weight testing machine (Figure 2), which converts the potential energy $E_{p}$ at the beginning of the fall,

(4) $$E_{p}=MgH,$$

into an axial crush velocity $v_{i}$ at the impact by conservation of linear momentum between the mass M of the falling ram and the mass $M_{t}$ of the upper tool part containing the compression platen,

(5) $$v_{i}=\eta M\sqrt{2gH}/M_{t},$$

In the above equation, g is the gravity acceleration and η is the efficiency, which accounts for the air resistance, friction sliding along the columns and type of collision between the falling ram and the upper tool part (Figure 2a). The mass M and the drop height H of the falling ram were adjusted to deform the crash boxes by approximately 1/3 of their initial length with an impact velocity $v_{i}≃16$ m/s. Finally, it is worth noting that the impact velocity $v_{i}$ is progressively reduced to zero by the absorption of energy.

As seen, the overall trend of the dynamic force-displacement curves is similar to that of the quasi-static tests (Figure 8a) but the peak force to trigger collapse increases from 89 kN to 115 kN, in case of the crash boxes assembled with “mortise-and-tenon” joints. Similar increase in the peak force is obtained for the resistance spot-welding joints.

### 3.4. Alignment of the “Mortise-And-Tenon” Joints

The crashworthiness performance of the new crash boxes with “mortise-and-tenon” joints raises the question of the alignment of the protrusions of the flat-shaped surface heads of the tenons above the adjacent sheet panel, after compression. Should the flat-shaped surface heads be collinear or perpendicular to the longitudinal axis of the crash box?

**Figure 9:**
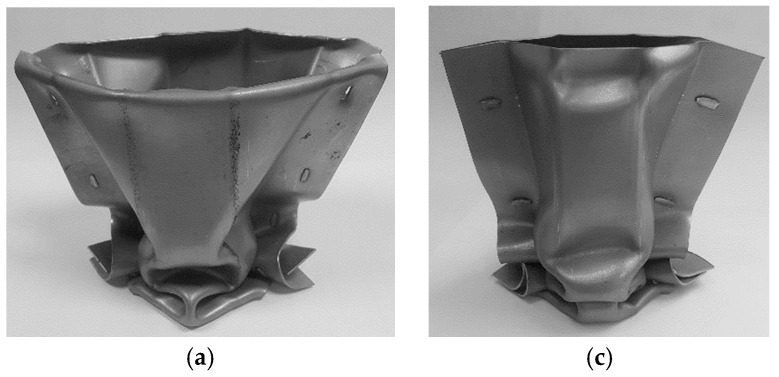

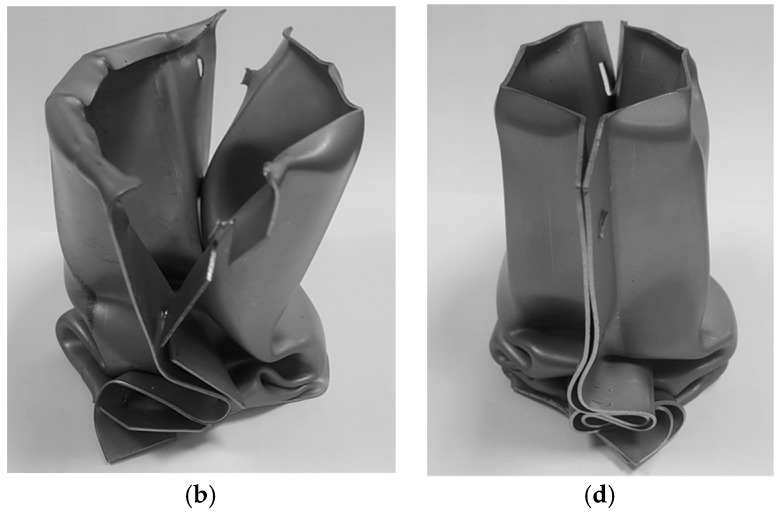
Thin-walled crash boxes with double-hat section assembled by sheet-bulk compression with “mortise-and-tenon” joints after axial crushing: The flat-shaped surface heads of the tenons are (**a**) collinear (front view), (**b**) collinear (side view), (**c**) perpendicular (front view), and (**d**) perpendicular (side view) to the longitudinal axis of the crash box.

Experiments performed by the authors revealed that the flat-shaped surface heads must be perpendicular to the longitudinal axis of the crash boxes because if they are collinear, they are easily pulled-out during axial crushing, diminishing the overall performance of the crash box (Figure 9).

## 4. Discussion

The evolution of the force with displacement for the quasi-static and dynamic axial crush tests performed with the two different types of crash boxes allow concluding that the new proposed “mortise-and-tenon” joints can successfully replace resistance spot-welds. In fact, Figure 8 shows that the overall trend of the force-displacement curves is similar with peak forces to trigger collapse being higher in the dynamic crush tests. In case of the crash boxes assembled with “mortise-and-tenon” joints, for example, the peak values increase from 89 kN to 115 kN when dynamic conditions are applied.

The energy E absorbed by the crash boxes during the axial crush tests are obtained from the areas below the experimental force-displacement curves of Figure 8,

(6) $$E=\int_{0}^{\delta_{\max}}Fd\delta ,$$

where $\delta_{\max}=55$ mm is the maximum specified testing distance (refer to Figure 8).

**Figure 10:**
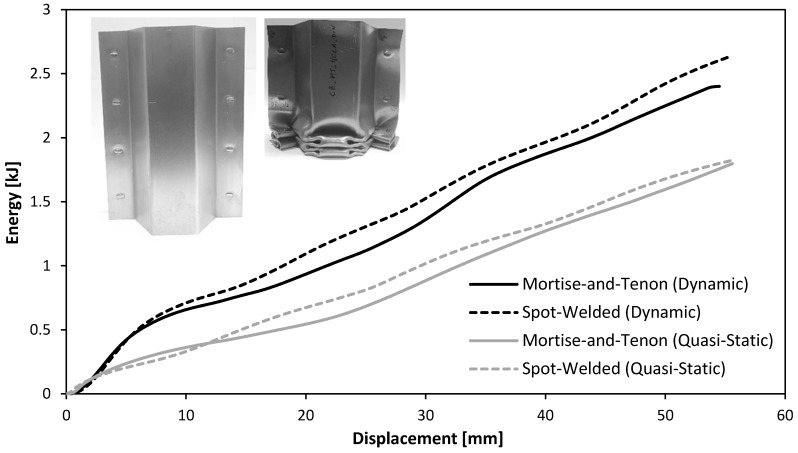
Experimental evolution of the absorbed energy with displacement for the axial crush tests of thin-walled crash boxes with double-hat section assembled by sheet-bulk compression with “mortise-and-tenon” joints and by resistance spot-welding: (**a**) quasi-static tests; (**b**) dynamic tests.

The results are shown in Figure 10 and allow concluding that the new type of crash box can absorb an overall level of energy similar to that of the resistance spot-welded crash boxes.

The maximum absorbed energy in the dynamic tests, is approximately 30% higher than in the quasi-static tests. This increase of crashworthiness performance with velocity is attributed to the strain-rate sensitivity of the HSLA 340 steel [16]. However, it is worth noting that the maximum absorbed energy in the dynamic tests is approximately 25% smaller than the total energy provided by the drop weight machine (3.2 kJ) because part of this energy is lost in the conversion of linear momentum between the mass M of the falling ram and the mass $M_{t}$ of the tool, in the elastic deformation of the tool and of the drop weight testing machine, and in the vibration of its different components.

**Figure 11:**
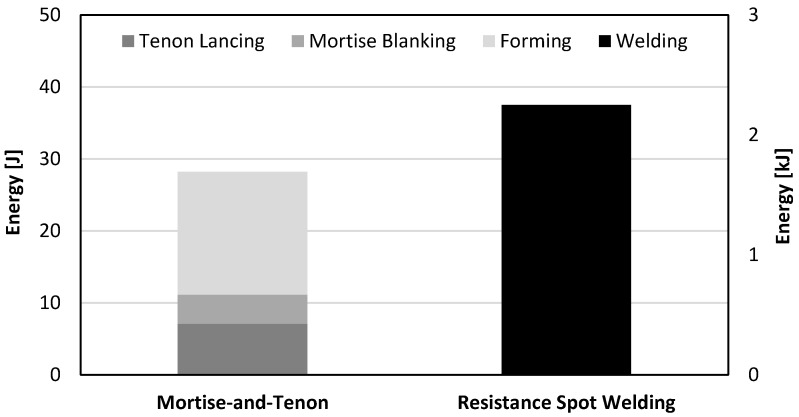
Energy required to produce “mortise-and-tenon” and resistance spot-weld joints. The energy required by the mortise-and-tenon joint is referred to the vertical left axis whereas that of the resistance spot-welded joint is referred to the vertical right axis.

Another result that is relevant from a manufacturing point of view is the total energy required to fabricate a “mortise-and-tenon” joint and a resistance spot-welded joint. From the experimental and numerical evolution of the force with displacement for the first and second stages of the sheet-bulk compression of the tenons it may be concluded that approximately 7 J and 10 J will be needed to accomplish both stages. Thus, by considering these values of energy as well as that required to perform the lancing operation, it is concluded that the total amount of energy to fabricate a “mortise-and-tenon” joint is a very small fraction of that required by a resistance spot-welded joint (Figure 11). This is an important advantage of the new proposed type of crash boxes regarding environmental friendliness.

## 5. Conclusions

The fabrication of crash boxes by sheet-bulk compression with “mortise-and-tenon” joints can successfully replace conventional production processes based on resistance spot-welding. The crash boxes with “mortise-and-tenon” joints can absorb the same amount of energy as those with resistance spot-welding joints in quasi-static and dynamic axial crush tests. They can also avoid the problems caused by residual stresses in the resistance spot-welding of panels made from dissimilar materials with different thicknesses.

The greater applicability of the new crash boxes comes with a disadvantage regarding productivity due to the multi-stage characteristics of the proposed joining by forming process. However, this disadvantage can be offset by the environmental friendliness resulting from the total required energy to assemble a crash box by sheet-bulk compression with “mortise-and-tenon” joints being a very small fraction (1.3%) of that required by resistance spot-welding.

## Figures and Tables

**Table 1:** Main characteristics of the fabrication processes that are commonly utilized to assemble thin-walled crash boxes with top-hat or double-hat shaped sections.

Process	Resistance Spot Welding	Adhesive Bonding	Self-Piercing Riveting	Clinching
**Materials**	Mostly steels. Metallurgical compatibility necessary for dissimilar metals	Engineering materials	Engineering materials with reasonable ductility and fracture toughness	Engineering materials with reasonable ductility and fracture toughness
**Coatings**	Thick metal and organic coatings are difficult	Coatings and lubricants must be cleaned	Organic coatings and lubricants can affect the properties of the joints	Organic coatings and lubricants can affect the properties of the joints
**Surface preparation**	None	Caution preparation	None	None
**Consumables**	Electrodes	Adhesives	Rivets (coatings needed for steel rivets)	None
**Environmental impact**	Sparks, fumes, and noise	Chemicals	Noise	Noise
**Aesthetics and geometry**	Indentation on both sides. Damage of coatings. Distortion and residual stresses due to thermal cycle	None	Flush in one side and protrusion on the other side	Hole in one side and large protrusion on the other side
**Performance**	High shear strength and medium peel strength	Low shear and peel strengths	High shear strength and medium peel strength	Medium shear strength and low peel strength
**Productivity**	High	Low (long curing time)	Intermediate	High (no pre-working required)

**Table 2:** Geometry and parameters of the thin-wall crash boxes assembled by sheet-bulk compression with “mortise-and-tenon” joints and by conventional resistance spot-welding.

Geometry		Sheet-Bulk Compression		Resistance Spot-Welding	
Thickness t	1 mm	Tenon width w	5 mm	Electric current	7.8 kA
Flange a	24 mm	Tenon length h	3.5–6 mm	Time (distributed in two pulses)	320 ms
Top section b	36.6 mm	Distance between joints d	40 mm	Force	3.3 kN
Angle α	120 degrees	Tapered punch angle β	~4°	Resulting nugget diameter D	5.4 mm
Length l	160 mm	Tapered punch length c	1.5 mm	Distance between welds d	40 mm
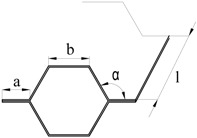		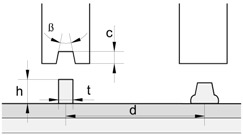		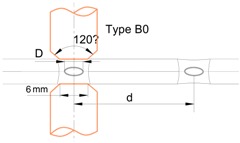	
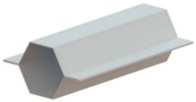		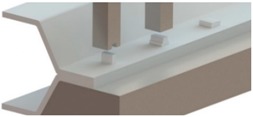		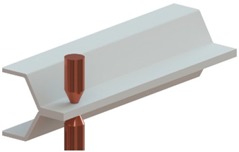	

**Table 3:** Summary of the quasi-static and dynamic axial crush testing conditions.

**Quasi-Static Axial Crush Tests**	
Equipment	Hydraulic testing machine
Velocity v	10 mm/min (1.7 × 10^−4^ m/s)
**Dynamic Axial Crush Tests**	
Equipment	Drop weight testing machine
Ram mass M	82 kg
Upper tool moving mass $M_{t}$	45 kg
Height of the fall H	4 m
Efficiency η	~60%
Velocity $v_{i}$	9.5 m/s

## References

[1] Baroutaji A., Sajjia M., Olabi A.-G. (2017). On the crashworthiness performance of thin-walled energy absorbers: Recent advances and future developments. Thin-Walled Struct..

[2] Yusof N.S.B., Sapuan S.M., Sultan M.T.H., Jawaid M., Maleque M.A. (2017). Design and materials development of automotive crash box: A review. Ciência Tecnol. Mater..

[3] Reyes A., Langseth M., Hopperstad O.S. (2002). Crashworthiness of aluminum extrusions subjected to oblique loading: Experiments and numerical analyses. Int. J. Mech. Sci..

[4] Jin S.Y., Altenhof W., Kapoor T. (2006). An experimental investigation into the cutting deformation mode of AA6061-T6 round extrusions. Thin-Walled Struct..

[5] Schneider F., Jones N. (2003). Influence of spot-weld failure on crushing of thin-walled structural sections. Int. J. Mech. Sci..

[6] Pan C.-L., Yu W.-W. (2002). Bending strength of hybrid cold-formed steel beams. Thin-Walled Struct..

[7] Abedrabbo N., Mayer R., Thompson A., Salisbury C., Worswick M., Riemsdijk I. (2009). Crash response of advanced high-strength steel tubes: Experiment and model. Int. J. Impact Eng..

[8] Mori K., Bay N., Fratini L., Micari F., Tekkaya A.E. (2013). Joining by plastic deformation. CIRP Ann. Manuf. Technol..

[9] Lee M.-H., Kim H.-Y., Oh S.-I. (2006). Crushing test of double hat-shaped members of dissimilar materials with adhesively bonded and self-piercing riveted joining methods. Thin-Walled Struct..

[10] Gronostajski Z., Polak S. (2008). Quasi-static and dynamic deformation of double-hat thin-walled elements of vehicle controlled body crushing zones joined by clinching. Arch. Civ. Mech. Eng..

[11] Bragança I.M.F., Silva C.M.A., Alves L.M., Martins P.A.F. (2017). Joining sheets perpendicular to one other by sheet-bulk metal forming. Int. J. Adv. Manuf. Technol..

[12] Pragana J.P.M., Silva C.M.A., Bragança I.M.F., Alves L.M., Martins P.A.F. (2018). A new joining by forming process to produce lap joints in metal sheets. CIRP Ann. Manuf. Technol..

[13] Alves L.M., Nielsen C.V., Martins P.A.F. (2011). Revisiting the fundamentals and capabilities of the stack compression test. Exp. Mech..

[14] Nielsen C.V., Zhang W., Alves L.M., Bay N., Martins P.A.F. (2013). Modelling of Thermo-Electro-Mechanical Manufacturing Processes with Applications in Metal Forming and Resistance Welding.

[15] SWANTEC Software and Engineering ApS SORPAS^®^2D Hybrid–Version 12.92. www.swantec.com.

[16] Strain Rate Sensitivity and Crash Modelling of High Strength Steels. https://thyme.ornl.gov/ASP_Main/matdata/matdata.cgi.

